# SOCIAL ENGAGEMENT AND DEPRESSIVE SYMPTOMS IN OLDER AFRICAN AMERICANS: A 6-YEAR CROSS-LAGGED PANEL ANALYSIS

**DOI:** 10.1093/geroni/igz038.2770

**Published:** 2019-11-08

**Authors:** James Muruthi, J Tina Savla

**Affiliations:** 1 University of Oregon, Eugene, Oregon, United States; 2 Virginia Tech, Blacksburg, Virginia, United States

## Abstract

Although previous studies have extensively investigated the cross-sectional relationship between social engagement and depressive symptoms in late life, longitudinal studies have produced mixed results. Furthermore, studies on the associations between these two concepts among aging African Americans are few. Using a sample of 1688 older African Americans adults from waves 1 and 7 of the National Health and Aging Trends Study (60% women; Average age = 77 years), the present study investigates the longitudinal associations between social engagement (an index from scores on visiting friends and family, attending religious services, attending religious services, participating in group activities, and going out for enjoyment) and depressive symptoms across seven years. Structural equation modeling was used to test cross-lagged relationships between the variables. Findings suggest that social engagement at baseline significantly predicted subsequent depressive symptoms and social engagement. Depressive symptoms at baseline, however, were not significantly associated with subsequent social engagement. These findings suggest that low social engagement in older African Americans is directly associated with increased depressive symptoms over time, but not vice versa. The implications of these findings are discussed in relation to the barriers of social engagement for older African Americans and its effects on their mental health.

